# Foreign Summary

**Published:** 1838-09-12

**Authors:** 


### FOREIGN SUMMARY.

*Useful Application to Bed-Sores.*—A correspondent of the Bulletin Génerale de Therapeutique recalls to the attention of medical men a very excellent, and easily prepared, local application for those troublesome and distressing sores which are so apt to occur in bed-ridden patients.

It is unnecessary to allude to the frequency of this annoyance in certain cases of protracted disease, more especially of obstinate fevers, of phthisis, &c., and to the extreme difficulty of counteracting them. The late M. Autenreith, of Vienna, was much in the habit of using the thick sedementary deposit obtained by adding the liquor plumbi, drop by drop, to a strong decoction of oak bark, (in short, a *tannate of lead,*) as a topical remedy to bed-sores, with great success. The super-natant liquor being decanted off, the sediment is easily procured; it is then to be spread on linen, as we do with an ointment. The application to the abraded surface should be repeated every night and morning.

Dr. Tott, a countryman of M. Autenreith, has, of late years, used this remedy with very satisfactory effects. In some cases, where it did not seem to agree, he mixed a certain portion of the *tannate*, previously dried, with simple ointment, (two drachms to one ounce,) and he found that the sores often healed readily under the use of this cerate.

We can bear our testimony in favour of the good effects of this application to bed-sores. In our own practice we have prepared it by mixing together the liquor plumbi and the common tincture of kino.—*Lon. Med. Chirurg. Rev.*, *July*, 1838.

---

*Case of slow Poisoning by Oxide of Zinc.* By Dr. Busse, of Berlin.

A gentleman, aged forty-five, of regular habits and previously of good health, was, in 1825, attacked, without evident cause, with epilepsy, which continued to return at longer or shorter intervals. His physician recommended change of climate; and the three following years, during which there was no return of the disease, were spent in travelling, chiefly in Italy and France. But scarcely had he returned to Berlin when the fits again appeared. Various remedies were tried in vain; and the patient, happening to notice oxide of zinc, in combination with hyosciamus, recommended in Hufeland's Journal as a cure for epilepsy, resolved, without consulting any physician, to submit himself to this treatment. He secluded himself from society, and took daily, at an average, twenty grains of the oxide, till he consumed 3,246 grains, and would probably have continued his experiment to a fatal termination had not one of his relations insisted on being admitted to him, and by whom he was found in a most deplorable condition. Dr. Busse was immediately summoned, and found the patient of a pale earthy hue, wasted away, and almost idiotical: his tongue was thickly coated, the bowels were constipated, the inferior extremities cold and œdematous, the abdomen tumid, the superior extremities cold and shrivelled, and their skin dry like parchment; the pulse was about sixty, thready, and scarcely perceptible.

The oxide of zinc was immediately stopped, but not without some opposition on the part of the patient, and a purgative ordered. Light nutritive diet was allowed, with tonic and diuretic medicines; and under their use the patient rapidly improved, and soon regained his previous robust and healthy appearance, although he remained subject to epileptic attacks, which continued to recur every six or eight weeks. *Wochenschrift für die gesammte Heilkunde, from Brit. and For. Med. Rev.*, *July*, 1838.

---

The following graphic sketch of Sir Henry Halford, the "Court Physician," is from the pen of Dr. James Johnson.

"This distinguished physician—the son of a physician, Dr. Vaughan—was born in Leicester, Oct. 1766—and consequently is in his 72d year. He is a remarkable instance of the degree of energy, both mental and corporeal, which may be retained beyond the tenth septenniad! Sir Henry is just as active a practitioner at this moment, as he was twenty years ago, when we first became personally acquainted with him. Educated at Rugby and Oxford, Sir H. became an excellent classical scholar, and so retentive is he of all the grammatical niceties of the Greek and Latin languages, that, the last time we met him in consultation, we found him amusing his vacant *moments*—that is between the houses of his patients—by translating English ballads into Sapphic verse—verse which Horace would not have disowned in the Augustan age! Having contracted a matrimonial alliance with a lady of noble race, he soon became the fashionable physician of the day—probably more owing to the elegance of his manners, the extent of his learning, and the influence of his connexions, than to any superiority over his contemporaries in practical knowledge or pathological research. Indeed he got into high practice before the age of thirty—and never having been attached to any hospital, he could not, at that age, and especially at that era, have had much clinical experience, while pathology was almost unknown. Sir Henry presents one of the very few instances in this metropolis, of a physician penetrating into the very highest circle of practice, and, consequently, reaping the largest harvest of wealth, without being a medical officer of any hospital. Babington, Baillie, Curry, Warren, Pearson, Nevinson, Chambers, Elliotson, &c. &c., might be cited as illustrations. But Sir Henry outstripped all his compeers, with all the advantages of their clinical knowledge and patronage of pupils. We do not wonder at it. A notion prevails among those who are not acquainted with Sir Henry that, from his long course of attendance in the chambers of the high nobility, where imaginary are more numerous than real diseases, his practice was milk and water, and rarely active or decisive. This is an error. The worthy Baronet is by no means deficient of energy, where the case requires it—and his long experience enables him to detect the nature of a malady very readily, and grapple with it stoutly. It is very true that, for obvious reasons, this eminent physician has not been able to keep pace with the progress of pathology; and, consequently, he is defective in his diagnosis of diseases of the chest, whether cardiac or pulmonic. This deficiency, though it might be an awkward inconvenience in the wards or dead-house of an hospital, was of very trifling import in the rounds of private practice, where it was seldom suspected by the friends of the dead, and still more seldom demonstrated by the scalpel of the anatomist. But had this retardation in the march of pathology been ten times more patent than it was, it would have been amply compensated by the *tact* of this gifted physician, unequalled in any age or nation! We sincerely believe that, since the days of Esculapius himself, down to the present moment, no medical practitioner ever entered the room of sickness so well calculated to cheer the drooping spirits, raise the hopes, or inspire the confidence of the patient, as the physician whose biography is here undertaken! The smiling countenance, the courtly manners, the eloquent language, wearing the appearance, if not the reality, of the most sympathising friendship, the firm tone, the artful but judicious interrogations, the patient attention to garrulous details, the perfect mastery over every symptom of doubt, much less alarm—all combined, with numerous other traits of bearing and conduct which language cannot convey, to impress the sufferer with a conviction that, if the disease admitted of remedy, here was the man most qualified by nature and art to apply it. We have, on more than one occasion, heard patients remark, after Sir Henry had left the house, that they would almost as soon die under such a physician as recover under some others whom they could name! This, though the chief, is not the only qualification which Sir Henry possesses as a powerful passport to fame and favour in the eyes of the world. His resources are absolutely inexhaustible. Not only can he vary the daily physic, with surprising ease, but he can gratify the most languishing and fastidious appetite of the invalid with innumerable forms of diet, which would seem to have required half one's life to study and manipulate. By the magic wand of this accomplished physician, the most protracted, painful, and incurable maladies are converted into a kind of luxurious anticipation of daily relief from medicine, and hourly enjoyment of the palate. We are, at this time, attending an octogenarian, whose taper of life has been kept, for some months, from extinction by a glass of rum and milk, prescribed by Sir Henry, early in the morning, after which, the bed-ridden invalid falls into a delicious sleep of some hours, dreaming of early scenes, and awaking refreshed, with constant blessings on the head of the physician who ordered so delightful a cordial for the decaying frame! But Sir Henry's resources and endowments are not confined to physic or physics—they extend to metaphysics. The dying Christian would find in him a more cheering philosophy and religion than most of our divines would be able to convey to the spirit retiring from its earthly tabernacle to soar into other and unknown regions. Discourses have been delivered by the subject of our memoir, which would have been more appropriate in the mouths of prelates, than some of their harangues in the HOUSE OF THE LORD—leaving aside their orations in a house of many Lords.

With such natural talents, acquirements, and *tact*, is it wonderful that Sir Henry should have been the favourite physician of three successive sovereigns, with their courts, camps, senates, and aristocracy? The urbanity of his manners, the polish of his conversation, and the honourable etiquette of his conduct amongst his brethren, have rendered him a favourite consultant in all cases, and secured his professional popularity up to the eleventh septenniad of his life. We have met him when we were at "daggers drawn" with the College over which he had so long presided, and when our professional existence was in some danger from the anathemas of that once powerful, but now very harmless, body of medical aristocrats; yet never experienced the least deviation from friendly intercourse and professional equality. From Sir Henry Halford we never received or solicited either favour or patronage—and never will. We have drawn up the foregoing sketch of his professional character, from twenty years' observation of it, and without referring at all to the memoir of Mr. Pettigrew. We have neither extenuated the defects nor exaggerated the virtues of this distinguished physician, being perfectly independent of his frowns or favours; but only desirous of exhibiting the prominent traits of character which we have observed, without the slightest bias from medical politics or personal considerations.—*Medico-Chirurgical Review, July*, 1838.

---

*Luxation of the Scapular Extremity of the Clavicle, downwards. By* DR. TOURNEL.

This case is interesting, as being, apparently, with one exception, the first of the kind hitherto detailed; and as being one, also, the possibility of which has been questioned.

A soldier was thrown from his horse. The horse fell on its rider, and, on recovering itself, placed its foot on the front of his left shoulder, where was an ecchymosis of almost the exact shape of the horse's shoe. The pressure separated the scapula backwards. The clavicle remained attached to the sternum; but, its superior and inferior and coraco-clavicular ligaments having been torn, its external extremity slipped from its articular surface beneath the acromion. The accident was regarded at first as a dislocation of the humerus, but the true nature of it was ascertained by an examination in the following manner. The summit of the shoulder was grasped with one hand, resting on the acromion, whilst, with the other hand, it was ascertained, by various motions, that the axis of the humerus was in its ordinary direction. There was no bony projection in the axilla. The left arm was somewhat longer than the right; the elbow and all the rest of the limb were in contact with the lateral part of the trunk. Voluntary movements, and especially upwards, were impracticable: communicated movements were easy, and unattended with pain. The shoulder had lost its rounded form, and there was a depression outwards, beneath the acromion. The shoulder presented, in addition, two projections; one internal and superior, formed by the acromion; the other external and inferior, formed by the external extremity of the clavicle. There was no numbness or pain of the fingers. The summit of the left shoulder was much nearer the sternum than that of the right; and, when the finger was passed along the spine of the scapula from behind forwards, as far as its acromial extremity, it was not stopped by the clavicle. This had been perfectly recognised; and it disappeared, together with the sub-acromial depression, when, the knee having been placed between the two shoulders, they were both drawn backwards; but, when this traction was discontinued, the projection, formed by the external extremity of the clavicle, and the depression, were reproduced. The association of all these symptoms leaves no room to doubt that this accident was a dislocation of the scapular extremity of the clavicle, downwards. The reduction was easily effected by placing the knee against the vertebral column, and drawing the shoulders backwards. A cushion was then placed, and retained by bandages in the axilla. The arm pressing upon the cushion was kept applied to the trunk upwards and backwards, by Desault's bandage for fracture of the clavicle. Spirituous lotions were applied to the shoulder. The fore-arm was placed in a sling, and the whole kept in position by a bandage passing round the body. The impatience of the soldier required the removal of this apparatus. Instead of it was substituted that of M. Flamant, which has the advantage of leaving the injured part uncovered. This consists of a grooved bag,* to the angles of which are sewn two rollers, and of a pad, which is placed, as above, in the axilla. The arm being placed in the bag so that the elbow corresponded to its middle angle, the roller, sewn to its anterior angle, was passed over the middle and dorsal part of the fore-arm, and continued in front of the chest. The other was continued over the back part of the arm, and crossed the former over a thick compress placed upon the uninjured shoulder. The rollers were continued in these directions for two or three turns, crossing one another over the uninjured shoulder, and beneath the elbow of the opposite side. The remainder of the rollers was then passed round the trunk, in order to fix the arm. At the elbow, the bandage was kept in its situation by four tapes, two of which were sewn to the inner side, and two to the outer side of the sac in which the elbow rested. The whole apparatus was covered by a bandage of the trunk, and the scapulary of this bandage was employed to keep resolutive compresses applied to the injured shoulder. Notwithstanding his impatience, the soldier perfectly recovered, after thirty-two days' treatment. The first use which he made of his arm was to severely castigate his horse. He remains in his regiment, and experiences neither pain in the shoulder nor difficulty in the movements of the arm.

In the "Ephémérides Nat. Cur.," is an account of a similar displacement. In both cases the cause was violence from above, and directly upon the scapular extremity of the clavicle.—*Archives Générales de Médecine, Décembre,* 1837, *from Brit. and For. Med. Rev.*, July, 1838.

*New method of reducing Dislocations of the Os Humeri. By* M. MALGAIGNE.

SOME months since, M. Malgaigne published, in the "Bulletin de Thérapeutique," an account of a method which he practised successfully for the reduction of dislocations of the os humeri. The following is an instance of the total failure of the old method, and the success of the new:

A stone-cutter, forty-one years of age, fell from a scaffold four feet high, and dislocated his shoulder. A surgeon, who saw him immediately after the accident, having made some attempts, pronounced that the reduction was accomplished. After some time, however, the patient, finding he did not regain the use of his joint, entered the hospital Saint Louis, under the care of M. Jobert. A dislocation of the os humeri into the axilla was easily recognised—now twenty-three days after the accident. M. Jobert proceeded to attempt the reduction by the usual method of extension and counter-extension; the arm carried in the horizontal direction. The first, second, and third attempts were equally futile, the pain increasing in proportion to the efforts at reduction. The method of M. Malgaigne was then adopted. An assistant stood upon a table close to the seat of the patient, placed his foot upon the left shoulder to make counter-extension, and pulled with his two hands the dislocated arm raised to a nearly vertical direction. The reduction took place immediately almost without effort, and, above all, with very little pain. M. M. reports a case in which he succeeded after twenty-one days. These cases seem to point out the truth of his assertion, that the cicatrization of the broken ligaments, and the formation of the new capsule, is not perfectly finished till the expiration of twenty-five or thirty days.—*Bulletin Général de Thérapeutique, Avril,* 1838—*Ibid.*

*Observations on a method of employing the Ammoniaco-Nitrate of Silver, as a test for demonstrating the presence of very minute quantities of Arsenic.* By THOMAS STEWART TRAILL, M. D.

"I NOW send the promised statement of the method of applying ammoniaco-nitrate of silver, as a test for the detection of minute quantities of arsenic, which I lately had the pleasure of showing to you. It is on the principle of Dr. Wollaston's microscopic, chemical investigations, and has been long employed by me as a mode of exhibiting the great delicacy of this test. I prefer it to the method of your ingenious correspondent, Dr. Murray, because it enables the operator to judge of the nature of the precipitate; as, for instance, whether it separate in flakes, or in uniformly minute particles diffused through the fluid.

I put a drop of the suspected liquid on a plate of clear glass, and near it another of the test; then join them by means of a glass rod, without completely mingling them. In the experiments exhibited to you, the liquid employed was a solution of one grain of arsenic boiled in 1000 grains of water, and by adding determinate proportions of water to portions of this liquid, any fractional part desired was readily obtained.

The following are the results of the experiments which you saw.

1. With $\frac{1}{1000}$ of a grain of arsenic, the test afforded a rich, yellow, flaky precipitate, which on subsidence left the liquid clear.

2. With $\frac{1}{2000}$ of a grain, the precipitate was also very distinct to the naked eye.

* Sac en forme de gouttière.

3. With $\frac{1}{4000}$ of a grain, the character of the precipitate was distinctly seen by the naked eye.

4. With $\frac{1}{8000}$ of a grain, the precipitate was still distinct to the eye.

5. With $\frac{1}{10000}$ of a grain, attentive observation could still trace the character of the precipitate without a lens.

6. With $\frac{1}{12000}$ of a grain, a lens of moderate power enabled me to observe the yellow flakes in a clear liquid.

7. With the $\frac{1}{16000}$ part of a grain, cloudiness was observed, but I could not be positive as to the shade of colour.

In all these experiments, it aids the eye much to place the glass plate upon some dark ground, such as the sleeve of a coat. There is no risk of confounding this precipitate with the phosphate of silver; for none of the phosphoric salts afford precipitates with the ammoniaco-nitrate of silver, though they do with the simple nitrate.

The test now mentioned is far superior in delicacy to the ammoniaco-sulphate of copper; though I have tried Dr. Murray's mode of applying it, which is certainly an improvement on the common method.

I may remark, that Dr. Murray seems greatly to have underrated the delicacy of the test by reduction. I have repeatedly, by drawing out the stem of a ball-tube to a capillary bore, after the introduction of the sulphuret of arsenic and dry soda-flux, obtained distinct metallic crusts, that did not weigh $\frac{1}{200}$ of a grain; and would always prefer the reduction process to any other, if the quantity were sufficient for this test."—*Ed. Med. and Surg. Journ.*, July, 1838.

### LITHOTRITY,

### IN A CASE WHERE THE STONE ADHERED TO THE BLADDER.

A MAN, named Cotte-Boutaillat, aged 57, of strong constitution and sanguine temperament, was admitted into the hospital of the Hôtel des Invalides on the 18th of June, 1837. In the course of the year 1836 (he did not recollect the period more precisely,) he began to experience a pricking at the end of the penis, extending along the urethra, every time he made water.

Before this period he had always made water with ease, and had never experienced any retention of urine, either complete or incomplete; nor had he ever remarked any change in the qualities of the urine. Two months afterwards, he passed bloody urine several times, particularly after long and tiring walks. He was then obliged to make water oftener than formerly.

A year after the appearance of the first symptoms, Cotte began to have a sense of weight in the fundament; and he was now obliged to make water every quarter of an hour.

The desire of voiding the urine became more frequent and more urgent the oftener it was satisfied, because, in consequence of the entire expulsion of the urine, the stone became in immediate contact with the mucous membrane of the bladder, and thus caused the desire of voiding the urine when there was not a drop to be expelled. At the same time, the call to go to stool was felt more frequently than usual.

Cotte came under the care of M. Larrey for violent hematuria. He had all the symptoms of stone in the bladder, yet none could be found on sounding; so that M. Larrey confined himself to treating the hematuria with emollient and hygienic remedies.

At a later period the patient was still in the hospital, when the surgical cases were put under the care of M. Pasquier, jun., the principal surgeon.

Cotte was still in the same state, and the hematuria frequently occurred. M. Pasquier, like his predecessor, recognised all the rational symptoms of calculus; but his first attempts at sounding were not more fortunate than M. Larrey's, and no stone was found.

On the 8th of November, however, M. Pasquier, succeeded in striking the calculus with a silver sound, and proposed to break it down the following day.

The following was the state of the patient:—Urine of good appearance; occasional hematuria, sometimes demanding the vigorous use of emollients; no suffering while in bed, but, if he walked, there were pains in the hypogastrium, in the fundament, and the extremity of the penis, followed by bloody urine; there was also a marked alteration in his general health. On the 9th of November the patient still felt fatigued by the examination of the previous day, and the breaking down of the calculus was put off till the following one.

On the 10th the *lithotriteur* was introduced without much difficulty. The operator made unsuccessful attempts for a quarter of an hour to seize the calculus, which, nevertheless, he touched every time he opened the branches of the instrument. M. Pasquier being prepossessed by the supposition that the calculus was at the fundus of the bladder, as it is in the immense majority of cases, had not sufficiently examined the other regions of the bladder, when he perceived that he had to do with an adherent and suspended calculus.

Its situation was accurately determined during this sitting, and the operator ascertained that it was suspended to the anterior and superior region of the bladder, above and behind the neck; it occupied the median line, and extended more to the right than the left. It was of more than a middle size, and its free surface, which was rough, was touched by the instrument; hence it was not encysted. It was very solid, and was supposed to consist of uric acid.

The operator being once satisfied that it was an adherent calculus (a rare anomaly,) resolved to proceed no farther that day; partly, because the patient was exhausted, and partly, because the instrument was not sufficiently curved to seize the stone.

*November 11th.*—The patient was suffering from fever, with pain in the hypogastrium; frequent desire of voiding the urine, which was reddish; and headach. The face was red and puffy. These symptoms were evidently caused by the prolonged examinations of the preceding day; suitable treatment was adopted, and the operation was put off.

*November 12th.*—The patient is better; the pulse is less frequent; the face less red and puffy; the headach is gone; there is less sensibility in the hypogastrium; the urine is limpid, but the desire of voiding is still frequent.

*November 13th to December 29th.*—The state of the patient continually varied from better to worse, so that delay was thought prudent.

*December 30th.*—Cotte's health now permitted a fresh examination of the bladder, which confirmed the former diagnosis both as to the situation and size of the calculus; it appeared to be large and very hard. M. Pasquier settled and described the manœuvre which would be requisite in order to catch the stone with the branches of the instrument.

*December 30th.—First sitting.* The instrument was easily introduced, and the stone was immediately seized. M. Pasquier tried to detach it, even at the risk of bringing away a portion of the mucous membrane; and, in order to do so, he gave the instrument a twisting movement, which he repeated several times; for he justly thought that the trituration of the calculus would be much more easy when it had fallen into the fundus. This manœuvre succeeded completely; a considerable calculous mass fell into the fundus of the bladder, and M. Pasquier ascertained that it was composed of two calculi, which were joined, or at least contiguous.

He then proceeded to break down the two calculi, in which percussion was necessary; but pressure was sufficient to triturate the secondary fragments.

A small quantity of stone was brought away, partly by the lithotritizer, and partly by the *videur;* and these fragments being joined to what was passed with the urine (which was carefully filtered,) made a mass about the size of a hazel-nut.

No bad symptoms came on, and the patient continued in a very satisfactory state until January 5, 1838, when the bladder was again examined with the jointed sound. No more detritus was found in the fundus of the bladder, but it was ascertained that a considerable portion of the calculus was still adhering. The bladder, wearied by these examinations, contracted spasmodically, and at the same moment the adherent calculus struck the concavity of the sound.

*January 6th.—Second sitting.* The method of proceeding was the same as before, and several portions of the adherent calculus were broken off. The quantity of stone extracted was equal to the first. The patient was comfortable during the rest of the day, but in the course of the night he was inconvenienced by a fragment of the stone wedged in the orifice of the neck of the bladder.

*January 7th*—M. Pasquier, when he came to see the patient, pushed the fragment back into the bladder by injections of tepid water.

*January 8th.—Third sitting.* The bladder was examined with the jointed sound, and no fragments were found in the fundus; the adherent portion alone remained, and extended chiefly to the right of the neck. It was necessary to have recourse to the *brisecoque* to divide it; several fragments were detached, and easily broken down. The *videur* was introduced, and assisted the exit of the fluid contained in the bladder; a great quantity of dust was suspended in the fluid. The mass of calculus obtained was equal to the preceding ones.

*January 9th.*—Tepid water was injected, and more detritus was obtained. The state of the patient continued to be satisfactory.

*January 10th.—Fourth sitting.* Two pieces were detached from the calculus, and easily broken down. Tepid water was injected, but the quantity of detritus which followed was smaller than at the preceding sittings.

*January 11th.—Fifth and last sitting.* The manœuvres to separate the portion of the stone still adhering to the bladder were repeated, and were supported by an assistant, who used moderate compression upon the hypogastric region. They completely succeeded, and the remaining portion of the calculus fell into the fundus of the bladder, where it was easily broken down. A tepid injection was followed by the exit of a very small quantity of detritus.

Up to the 22d of January inclusive, no bad symptoms, either general or local, had occurred; the state of the patient was very satisfactory; and the urine gradually became limpid.

On the 23d of January, a bit of gravel was passed in making water. M. Pasquier, on visiting the patient, threw up an injection, and then examined the bladder with the jointed sound; but not an atom of detritus was detected. He then pointed out the necessity of watching the state of the bladder for some time.

*January 24th to 30th.*—The examinations were repeated several times, and the bladder was always found perfectly clear; Cotte, therefore, was now considered as entirely freed from the stone.

To finish the case, we will add, that the calculus was almost entirely composed of uric acid, and showed, on analysis, only some slight traces of oxalate of lime. After every sitting the patient experienced a little feverishness, which rarely lasted till the following day; and at the same time there was sensibility in the hypogastrium, with prickings in the glans, and frequent desire of making water, which soon went off.

Baths were often employed, especially the bidet, as well as clysters, which were at various times simple, emollient, narcotic, and antispasmodic. He also had poultices upon the hypogastrium, with or without laudanum; and took infusions of chamomile, mallow, or violet, sweetened. The regimen was severe, and was often modified according to the indications.

But what contributed above all to the success of the operation, were the attentions of every kind, hygienic and therapeutic, which are lavished with such exactness upon all the patients under M. Pasquier's care, and which, unquestionably, go incalculably far towards the cure of a patient, particularly when the case requires long and assiduous treatment, as in the subject of this article.—*Gazette des Hôpitaux, from Lon. Med. Gazette, June*, 1838.

---

We have had occasion, says Madame Boivin, to see fine young women, who exhibited all the characters of maidenhood, extremely subject to catamenial irregularities, and each labouring under a scirrhous affection of the mammæ. Three of these patients were operated upon by the Baron Dubois: their respective ages were 22, 25, and 38.

